# Update on Prostate Cancer Diagnosis, Prognosis, and Prediction to Response to Therapy

**DOI:** 10.3390/cells10010020

**Published:** 2020-12-24

**Authors:** Rodolfo Montironi, Alessia Cimadamore, Antonio Lopez-Beltran, Liang Cheng, Marina Scarpelli

**Affiliations:** 1 Section of Pathological Anatomy, School of Medicine, United Hospitals, Polytechnic University of the Marche Region, 60126 Ancona, Italy; a.cimadamore@staff.univpm.it (A.C.); m.scarpelli@univpm.it (M.S.); 2 Department of Surgery, Cordoba University Medical School, 14071 Cordoba, Spain; em1lobea@gmail.com; 3 Department of Pathology and Laboratory Medicine, Indiana University School of Medicine, Indianapolis, IN 46202, USA; liang_cheng@yahoo.com

The wide range of novelties reported in this Special Issue of the journal *Cells* on prostate cancer (PCa) diagnosis, prognosis, and prediction to response to therapy, has led us to a series of considerations related to a better understanding of the current and future role of effective molecular biomarkers in individual patients with PCa [1,2,3,4,5,6,7]: (1) Digital pathology, including multiplexing; (2) Clinical translation of genetic information; (3) Clinical significance of liquid biopsies; and (4) Multi-criteria decision making and information fusion.

## 1. Digital Pathology

Tissue-based diagnostics is becoming increasingly digitalized to expand the great potential of personalized medicine, including the administration of new therapeutic approaches [8]. “Therefore, the daily task for pathologists is changing drastically and will become increasingly demanding in order to take advantage of the development of modern computer technologies” [8]. The role of histopathologists has evolved from describing the morphology of a lesion to that a “gatekeeper” for novel prognostic factors as well as effective treatment options. “This is possible based on the retrieval and management of a wide range of complex information from tissue or a group of cells and associated meta-data” [8] (See below: *Multi-criteria decision making and information fusion*). Self-learning and intelligent software can support histopathologists in their efforts to make clinically appropriate decisions in patients with PCa on the basis of the accurate quantification of multiple molecules and of surrogate biomarkers, together with contextual information and spatial relationships obtained from virtual slides and multiplexed images [8].

### Multiplexing

“For every new drug in development, appropriate biomarker assays that can be applied to formalin-fixed paraffin-embedded tissue samples are developed and then tested for correlation with clinical responses and efficacy” [9]. Each of the tissue biomarkers that is predictive of clinical response to a specific drug will be included in the routine practice of histopathologists [10].

**Figure 1:**
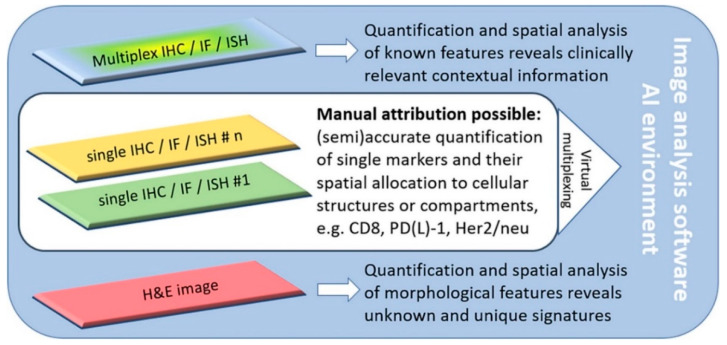
Software-assisted decision support in digital histopathology. Most current problems in routine histopathology can be solved with software-guided image analysis tools. The increasing emergence of complex multiplex analysis allows for the consideration of the tumor heterogeneity and the spatial relationships of various markers. If single-slide multiplexing is not possible due to inconsistent antigen-retrieval or disparate staining protocols, virtual multiplexing is also an option. Novel artificial intelligence (AI) solutions even allow novel and unique signatures from H&E slides to be revealed. Reproduced with permission from *J. Pathol.* **2020**, *250*, 685–692, doi:10.1002/path.5388 [8].

A major problem for histopathologists is that there are multiple biomarkers and several analytical assays to perform in order to obtain the best comprehensive profile for each patient [9] (Figure 1).

Multiplexed phenotyping assays of formalin-fixed paraffin-embedded (FFPE) tissues are “in the early phases of deployment and under very active development” [9]. These include multiplexed immunofluorescence. This is based on immunostaining FFPE samples with several antibodies and with an equal number of fluorescently conjugated secondary antibodies, applied in a sequential manner, which involves imaging the immune-stained sections with a microscope with specific spectral filters or with a multispectral imager [10].

Image processing and analysis are then used to construct an image of the tissue in which the expression of each biomarker is individually detected and quantitated [11]. By using multiplexed immunohistochemistry with “high-resolution whole-slide tissue imaging and analysis”, it is possible to obtain the automatic classification of epithelial cells and glands in PCa, including the evaluation of androgen receptor and alpha-methylacyl-CoA expression at the cell level [11]. This approach allows for an accurate evaluation of biomarker expression in each cell; cell phenotyping, based on multiple biomarkers; cell number, according to phenotype, as well as the “geometric relationship among cells of identical or distinct phenotypes” [9,10].

With multiplex staining on a single slide and high-resolution image analysis, machine-vision and deep-learning techniques offer diagnostic algorithms, including artificial intelligence that can be adopted for the precise diagnosis, prognosis and prediction to the response to therapy of PCa patients [8,9].

## 2. Clinical Translation of Genetic Information

Several groups of researchers are focusing their efforts on the translation of genomic data for clinical applications. For instance, several studies are currently investigating the androgen receptor signaling, MYC amplification, DNA-PKcs activity, ETS fusions and PARP, PI3K/mTOR/AKT pathway, etc. [12]. A significant challenge in the field of PCa is the low incidence of individual genomic alterations. “Enrichment for the more common mutations, such as androgen signaling, ETS fusions, and PI3K pathway activation, may allow enrollment into more traditional trials with random assignment between groups whereas rare or private molecular disease subsets will require studies based on these specific alterations” [12]. For each patient, repeat biopsies, usually at the time of tumor progression, are necessary to evaluate the development of new and changing subclones as well as mechanisms of resistance in order to make the appropriate changes in the treatment [12].

The use of genomic data in patients with metastatic castration-resistant PCa (mCRPCa) for the purpose of choosing the appropriate treatment is becoming a reality. Genomic data show that most of patients with mCRPCa harbor clinically actionable molecular alterations. “Clinical trials based on genomic data are now available, but more are in development. Nonetheless, many important challenges remain” [12].

For instance, obtaining viable tumor tissue from osseous locations is a significant challenge, although some researchers have shown a relatively high success rate in this field [12]. The genomic analysis of cell-free DNA and circulating tumor cells (liquid biopsy) is an attractive alternative to sequential tissue biopsies [12] (See below: *Clinical significance of liquid biopsy*).

Sequencing technologies show limitations because they do not fully account for the effect of tumor epigenetics or for the role of the tumor microenvironment [12]. Additionally, tumor heterogeneity is a significant challenge. The molecular characterization of PCa shows a certain degree of complexity. However, our understanding of such complex alterations is much greater.

## 3. Clinical Significance of Liquid Biopsy (Blood and Urine)

Compared to traditional diagnostic tissue-based procedures, liquid biopsy offers a promising perspective for PCa diagnosis and monitoring, showing several advantages. “Quick and minimal risk technique, minimal invasiveness, less expensive procedure are among the advantages of liquid biopsy compared to tissue” [9]. This allows the more accurate tracking of tumors and their mutations over a period of time during the follow-up [9].

The liquid biopsy of circulating tumor cells (CTCs) has been validated as a prognostic tool in patients with PCa, and more generally, with a variety of genitourinary cancers. This is based on the ability of CTCs to mirror tumor heterogeneity and the possibility “to combine the genetic and transcriptomic status of single CTCs with epigenome analyses” [13]. For instance, the epigenome analysis of the promoter of three genes regulating epithelial-to-mesenchymal transition (EMT) was applied to single CTCs from the blood samples of patients with metastatic castration-resistant PCa. A higher level of methylation in the promoter of the microRNA-200 family was observed in prostate CTCs, thus pointing out the tumor-specific activation of EMT-associated genes in association with metastatic spreading [14].

Voided urine is increasingly adopted for the diagnostic, prognostic, and predictive evaluation of genitourinary tumors, particularly PCa, by quantifying, for instance, cancer-associated RNA transcription and methylation [9,13,14]. “A risk score based on the mRNA profiling in urine, combined with traditional clinical risk factors, has been validated to identify patients with high-grade PCa (Gleason score ≥ 7) on prostate biopsy” [14].

## 4. Multi-Criteria Decision Making and Information Fusion

The process of merging multiple data originating from various sources is defined as multi-criteria decision making and information fusion [9,15,16,17]. Such an approach is often adopted in fields, including robotics, when a great amount of data needs to be contextualized as high-level information and interpreted [16]. The resulting information, including the diagnostic and therapeutic approaches and decisions, when applied to PCa, for instance, examined with large format histology and whole-slide imaging [17], and with multiple biomarkers derived from tissue, urine, and blood samples, is far more accurate than when the various sources are evaluated separately and individually [18]. There is still some kind of concern related to the application of such new tools, including software design, validation, storage as well as costs.

## 5. Conclusions

“The identification of effective biomarkers has become a major focus, mainly due to the necessity of selecting potentially responsive patients and to improve their outcomes, as well as to reduce the toxicity and costs related to ineffective treatments” [9]. Research on PCa is accompanied by the development of complex emerging techniques [9]. The pathologist has to integrate information from the pathological evaluation with the data from different sources to achieve a final diagnosis, prognosis and prediction to the response to therapy [4]. The ultimate issue is to improve our understanding of genomics in order to identify actionable targets as well as to develop novel treatments for patients with advanced PCa to extend survival and possibly provide cure [5].

## Abbreviations

PCa	Prostate Cancer
AKT	Protein kinase B
AMACR	alpha-methylacyl-CoA
PI3K	fosfoinositide 3-chinasi
mTOR	mammalian target of rapamycin
ETS	erythroblast transformation specific
PARP	Poly (ADP-ribose) polymerase
NA-PKcs	DDNA-dependent protein kinase, catalytic subunit
MYC	Myelocytomatosis oncogene cellular homolog
mCRPCa	metastatic castration-resistant prostate cancer
CTCs	circulating tumor cells
EMT	epithelial-to-mesenchymal transition
